# Ten Commandments of Off-Pump Coronary Artery Bypass Surgery

**DOI:** 10.1016/j.atssr.2025.09.027

**Published:** 2025-10-23

**Authors:** Shahzad G. Raja

**Affiliations:** Department of Cardiac Surgery, Harefield Hospital, London, United Kingdom

## Abstract

**Background:**

Off-pump coronary artery bypass (OPCAB) surgery offers distinct advantages over conventional on-pump techniques, including reduced inflammatory response, lower stroke risk, and improved outcomes in high-risk patients. Despite these benefits, concerns persist regarding graft patency, incomplete revascularization, and technical complexity, limiting widespread adoption.

**Methods:**

A focused literature review was conducted using PubMed-indexed sources published between 2000 and 2025. The medical subject heading term “coronary artery bypass, off-pump” was used in combination with keywords including “hemodynamic optimization,” “conversion,” “graft patency,” and “surgical training.” Only peer reviewed randomized trials, meta-analyses, guideline-based reviews, and expert consensus articles were included. Findings were synthesized to define 10 core principles for safe and reproducible OPCAB practice.

**Results:**

The “Ten Commandments” address key domains: patient selection, contraindication management, team readiness, stabilization techniques, hemodynamic control, complete revascularization, conversion avoidance, skill acquisition, technical refinement, and evidence-based advocacy. Each principle is supported by clinical data and practical strategies. Innovations, such as intracoronary shunting, advanced stabilizers, and artificial intelligence-guided decision-making, enhance procedural reliability and outcomes.

**Conclusions:**

OPCAB can be performed safely and effectively with structured preparation, multidisciplinary collaboration, and adherence to best practices. By embracing these commandments and integrating emerging technologies, surgical teams can overcome barriers to adoption, refine intraoperative techniques, and deliver individualized coronary revascularization with superior long-term outcomes.

**In Short**

▪ Off-pump coronary artery bypass (OPCAB) is a safe and effective alternative to conventional on-pump surgery, particularly in high-risk patients.
▪ Optimized hemodynamic management and surgical precision are critical to procedural success and long-term outcomes.
▪ Adherence to the “Ten Commandments” of OPCAB fosters enhanced adoption by ensuring procedural consistency, safety, and reproducibility.

Off-pump coronary artery bypass (OPCAB) has evolved from a niche technique into a validated alternative to conventional on-pump coronary surgery, particularly in patients with elevated operative risk. By avoiding cardiopulmonary bypass, OPCAB aims to mitigate systemic inflammatory responses, reduce perioperative morbidity, and accelerate recovery.^1^^,^^2^ Despite its technical complexity, OPCAB has demonstrated consistent safety, efficacy, and long-term durability when performed with surgical discipline and precision.^3^

This mini-review introduces a structured framework—the “Ten Commandments” of OPCAB—designed to codify essential operative principles that underpin procedural success. These commandments reflect accumulated clinical experience, evidence-based practice, and a commitment to reproducibility, offering a pragmatic guide for enhanced adoption and consistent outcomes in contemporary cardiac surgery.

## Material and Methods

A focused literature review was conducted using PubMed-indexed sources published between January 2000 and July 2025. The medical subject heading term “coronary artery bypass, off-pump” was used in combination with keywords including “hemodynamic optimization,” “conversion,” “graft patency,” and “surgical training.” Titles and abstracts were screened for relevance to operative technique, intraoperative challenges, clinical outcomes, and educational strategies. Full-text articles were reviewed to extract recurring themes and evidence-based insights pertinent to OPCAB practice.

Drawing on this evidence base, the Ten Commandments of OPCAB were formulated through thematic synthesis and reflective analysis. Each commandment represents a distilled operative principle, grounded in clinical reasoning and informed by personal experience in surgical education, peer review, and institutional practice. The framework is intended to codify best practices, promote reproducibility, and support enhanced adoption of OPCAB in contemporary cardiac surgery.

## Results

### The Ten Commandments of OPCAB

Through thematic synthesis of the literature and reflective clinical analysis, 10 foundational principles were identified to guide safe, reproducible, and effective OPCAB surgery. These principles—framed as “commandments”—emphasize patient selection, operative discipline, team preparedness, and strategic advocacy. They are summarized in Table 1 and elaborated below with supporting rationale.

**Table 1:** Ten Commandments of Off-Pump Coronary Artery Bypass Grafting

Commandment 1: Thou shalt select thy patients with prudence
Commandment 2: Thou shalt honor the contraindications
Commandment 3: Thou shalt prepare thyself and thy team meticulously
Commandment 4: Thou shalt stabilize the beating heart skillfully
Commandment 5: Thou shalt revere hemodynamic
Commandment 6: Thou shalt perform complete revascularization
Commandment 7: Thou shalt avoid emergency conversion
Commandment 8: Thou shalt negotiate the learning curve systematically
Commandment 9: Thou shalt refine thy techniques for thy patient's safety
Commandment 10: Thou shalt address the concerns of thy critics

Each commandment reflects a critical domain of OPCAB practice. Commandments 1 through 3 emphasize the importance of preoperative planning, prudent patient selection, and meticulous team preparedness. Commandments 4 through 6 focus on intraoperative technique, including effective stabilization of the beating heart and vigilant management of hemodynamics. Commandments 7 through 9 address risk mitigation, systematic negotiation of the learning curve, and ongoing refinement of surgical techniques to enhance patient safety. Finally, Commandment 10 advocates for thoughtful engagement with the broader surgical community, encouraging constructive dialogue and evidence-based adoption. Collectively, these tenets form a cohesive framework for procedural excellence and institutional reproducibility in OPCAB surgery.

## Comment

OPCAB surgery has evolved into a technically sophisticated and evidence-supported modality for myocardial revascularization. Its success hinges on a constellation of interdependent factors—meticulous patient selection, precise stabilization, vigilant hemodynamic management, complete revascularization, and structured training. Despite its documented advantages, OPCAB remains underused, often due to misconceptions, institutional variability, and the steep learning curve associated with operating on a beating heart.^4^ This discussion synthesizes the core principles that underpin successful OPCAB practice and explores future directions to enhance its adoption and outcomes.

### Patient Selection and Risk Stratification

Successful OPCAB begins with meticulous patient selection. Suitability depends on coronary anatomy, ventricular function, comorbidities, and tolerance to cardiac displacement. OPCAB offers the greatest benefits in high-risk populations—those with renal impairment, advanced age, cerebrovascular disease, or left ventricular dysfunction.^5^ However, limiting OPCAB to only high-risk cases may obscure its efficacy due to confounding patient-related complications. Extending its use to lower-risk patients fosters institutional proficiency, improves technical precision, and validates safety across the spectrum of coronary disease.

Multidisciplinary collaboration is essential.^6^ A cohesive heart team—comprising surgeons, anesthetists, interventional cardiologists, and perfusionists—ensures comprehensive evaluation. Risk stratification tools, such as European System for Cardiac Operative Risk Evaluation 2011 revision (EuroSCORE II) and The Society of Thoracic Surgeons models, guide decision-making.^7^ Dedicated OPCAB-trained anesthetists enhance intraoperative management, reducing conversion risk. This integrated approach improves procedural success and patient outcomes.

### Recognizing and Managing Contraindications

Despite its advantages, OPCAB is not universally applicable. Absolute contraindications, such as profound hemodynamic instability, severe ventricular dysfunction, or refractory arrhythmias, must be respected.^8^ These patients are better served with conventional on-pump coronary artery bypass grafting or on-pump beating heart coronary artery bypass grafting.

Relative contraindications, including extensive coronary calcification or diffuse small-vessel disease, require nuanced evaluation.^9^ Adjunct techniques, such as intracoronary shunting and enhanced stabilizers, can help navigate borderline cases safely. Individualized decision-making, supported by multidisciplinary input, ensures OPCAB is pursued in suitable candidates while maintaining contingency plans for those at risk.

### Surgical Readiness and Team Proficiency

OPCAB demands flawless execution without the physiological safety net of cardiopulmonary bypass.^10^ Preparation begins with comprehensive assessments, strategic imaging, graft planning, and a precise surgical roadmap. Advanced imaging—coronary angiography and echocardiography—guides graft selection and anastomotic strategy. Intraoperative flow assessment tools verify graft patency and reduce complications.

Team coordination is paramount. Surgeons, anesthetists, and operating room staff must be proficient in OPCAB-specific techniques. A well-prepared team enhances procedural reliability and patient safety.^10^

### Heart Positioning and Stabilization Strategies

OPCAB requires mastery of stabilization techniques to minimize cardiac motion while preserving perfusion. Suction stabilizers anchor coronary segments with minimal trauma. Device selection should match graft site anatomy.^11^ Effective myocardial exposure, aided by apical vacuum positioners or pericardial traction sutures (Supplemental Video 1), ensures access while maintaining stability.^12^ Intracoronary shunts protect perfusion during anastomosis.^13^ Even lateral wall revascularization, once considered challenging, is now feasible with refined techniques.

### Hemodynamic Optimization Strategies

Maintaining hemodynamic control is central to OPCAB success. Cardiac displacement and vessel occlusion can trigger hypotension, ischemia, and arrhythmias.^14^ Proactive volume management avoids excessive preload while preserving perfusion pressure. Vasoactive agents must be titrated to support oxygen delivery without increasing afterload.^15^

Gentle manipulation and effective stabilization minimize abrupt shifts.^16^ Continuous monitoring—transesophageal echocardiography and real-time flow assessment—provides actionable feedback.^14^ Close intraoperative communication ensures dynamic adjustments, reducing conversion risk and enhancing myocardial protection. Table 2 outlines key interventions across preoperative planning, anesthetic management, surgical technique, and intraoperative monitoring that collectively contribute to optimal hemodynamic control during OPCAB procedures.

**Table 2:** Key Interventions Described to Achieve Optimal Hemodynamics During Off-Pump Coronary Artery Bypass Grafting

Preoperative Optimization ○ Careful volume management to maintain adequate preload without excessive fluid loading. ○ Risk stratification using scoring models such as EuroSCORE II and STS risk algorithms. ○ Preoperative discussions with a multidisciplinary heart team to anticipate hemodynamic challenges. ○ A heart rate between 60 and 80 beats/min is generally preferred, because lower rates improve surgical precision while maintaining adequate myocardial perfusion. ○ Excessive tachycardia (90 beats/min) can increase myocardial oxygen demand and compromise stability.
Anesthesia Strategies ○ Using OPCAB-trained anesthetists proficient in managing hemodynamic fluctuations. ○ β-Blockers (eg, esmolol, metoprolol) can be used intraoperatively to achieve controlled bradycardia, reducing myocardial oxygen consumption and enhancing grafting accuracy. ○ Avoiding excessive sedation or volatile anesthetic agents that could depress myocardial function and compromise hemodynamics. ○ Judicious use of vasoactive agents (vasopressors or inodilators) to maintain coronary perfusion pressure. ○ Monitoring and adjusting preload to facilitate cardiac mobilization without inducing hypotension. ○ Continuous intraoperative assessment using transesophageal echocardiography for dynamic feedback.
Surgical Techniques ○ Gentle cardiac manipulation to avoid abrupt changes in preload and afterload. ○ Use of effective stabilization devices (suction or compression stabilizers) to minimize cardiac motion. ○ Strategic heart positioning with apical vacuum-assisted positioners or pericardial traction sutures. ○ Temporary epicardial pacing may be required in patients with pre-existing conduction abnormalities or excessive bradycardia to maintain a stable rhythm. ○ Atrial pacing can be useful for optimizing atrioventricular synchrony, ensuring effective preload and cardiac output during grafting. ○ Intracoronary shunts during distal anastomoses to preserve regional myocardial perfusion. ○ Avoidance of excessive traction or distortion that could precipitate ischemia. ○ Real-time flow assessment to ensure graft patency and mitigate intraoperative instability.
Intraoperative Monitoring and Adjustments ○ Continuous hemodynamic monitoring with real-time adjustments in response to fluctuations. ○ Collaboration between surgeons and anesthetists to proactively manage instability.

EuroSCORE II, European System for Cardiac Operative Risk Evaluation 2011 revision; OPCAB, off-pump coronary artery bypass; STS, The Society of Thoracic Surgeons.

### Completeness of Revascularization

Achieving full revascularization is essential for long-term success.^17^ Incomplete grafting correlates with recurrent ischemia and repeat interventions. Complex anatomies demand adaptive techniques. Intraoperative imaging verifies graft patency.^18^^,^^19^ Sequential anastomoses and composite grafting may be necessary for challenging targets. Lateral and posterior vessel access requires precise positioning and specialized stabilizers. Intracoronary shunting mitigates ischemia during distal anastomoses.^20^ These measures ensure comprehensive revascularization and durable outcomes.

Careful preoperative planning of the anastomotic order is crucial in OPCAB. Typically, grafting begins with vessels that receive collateral flow, whereas those providing collateral support are addressed last. Supplemental Table and the Supplemental File outline a grafting sequence with positioning, stabilization, and anatomical details to guide reproducible, hemodynamically stable, and effective coronary revascularization.

Left anterior descending artery exposure is achieved by placing a rolled swab under the heart with a pericardial traction suture pulled toward left hip, allowing stable access for anastomosis (Supplemental Video 2). Intermediate artery exposure requires gentle lateral displacement of the heart, aided by targeted suction stabilization and deeper traction on pericardial sutures to maintain hemodynamic stability (Supplemental Video 3). Posterior descending artery exposure involves pulling of a deep pericardial traction suture caudally and left of midline, enabling access to the posterior interventricular groove without compromising perfusion (Supplemental Video 4). Obtuse marginal artery exposure is facilitated by rightward heart elevation and incremental rotation without compression, with the stabilizer positioned to minimize motion and preserve coronary flow (Supplemental Video 5).

Chronic total occlusions are prioritized early to minimize ischemic burden. In cases where temporary occlusion induces hemodynamic instability, completing the proximal anastomosis first allows rapid restoration of perfusion once the graft is in place.

### Minimizing Emergency Conversion

Emergency conversion to cardiopulmonary bypass poses significant risks. It often results from hemodynamic collapse, ischemia, or technical failure. Preventive strategies are essential. Patient selection and risk stratification reduce conversion likelihood. Continuous monitoring detects instability early. Preoperative imaging guides procedural sequencing. Intracoronary shunts preserve perfusion.^21^

Structured conversion protocols must be in place. Early recognition and controlled transition to bypass prevent physiological shock.^22^ Institutional analysis of conversion cases refines future practice. Proactive management preserves procedural integrity and patient safety.^23^

### Systematic Negotiation of The Learning Curve

OPCAB mastery requires structured skill acquisition. Operating on a beating heart introduces unique challenges—stabilization, hemodynamic control, and precise anastomosis. Dedicated programs, including mentorship, simulation, and progressive case exposure, build competence.^24^

Familiarity with OPCAB-specific tools fosters fluency.^25^ Objective performance metrics, such as cumulative sum charts, ensure consistency. Mentorship in high-volume centers accelerates learning. Accreditation validates readiness.^26^ A systematic approach mitigates variability and secures OPCAB’s role in modern cardiac surgery.^27^

### Refining Surgical Techniques to Enhance Safety

OPCAB success hinges on mastering intraoperative techniques. Graft handling demands atraumatic manipulation and precision suturing to preserve endothelial integrity. Anastomotic accuracy, bleeding control, and ergonomic graft placement directly influence outcomes. Continuous refinement is essential. Intraoperative imaging, flow assessments, and postprocedural audits reinforce best practices. Risk-adjusted control charts guide performance improvement.^19^ Aortic “no-touch” techniques—using composite arterial grafts and in situ mammary arteries—reduce embolic risk and stroke incidence.^28^ Total arterial grafting enhances durability, especially in younger or complex patients.^29^ Internal mammary and radial arteries offer superior patency and resistance to atherosclerosis.

### Bridging Clinical Evidence and Critical Perceptions

Despite growing evidence, OPCAB adoption remains inconsistent due to concerns over graft patency, incomplete revascularization, and technical complexity.^30^ Early studies linked OPCAB to higher graft failure, but recent trials and meta-analyses show comparable outcomes with proper expertise.^31^^,^^32^ Long-term data highlight reduced stroke rates and improved recovery in high-risk patients.^30^

Concerns over incomplete revascularization have been mitigated by advances in stabilizer technology and training. Mastery of lateral and posterior grafting ensures complete revascularization. The narrative must shift from skepticism to evidence-based advocacy. Performance monitoring, accreditation, and dissemination of robust data will reinforce OPCAB’s role in modern cardiac surgery.^30^

### Integrating Artificial Intelligence and Future Research Directions

Artificial intelligence-assisted meta-analyses and registry-based studies offer powerful tools for synthesizing existing OPCAB data into actionable insights.^33^ Rather than repeating broad randomized trials, artificial intelligence enables high-resolution data integration across centers, extracting granular conclusions from prior studies. Automated data synthesis can guide precision-based improvements, refining techniques without the need for repetitive large-scale trials.^34^

As artificial intelligence evolves, its role in OPCAB will expand, transforming preoperative planning, intraoperative decision-making, and postoperative care into predictive, personalized, and data-driven processes.^35^^,^^36^ Embracing artificial intelligence allows cardiac surgeons to systematically enhance safety and efficacy, reinforcing OPCAB surgery’s place in modern coronary revascularization.

### Conclusions

The Ten Commandments of OPCAB provide a structured framework for best practices, emphasizing meticulous patient selection, hemodynamic control, stabilization mastery, complete revascularization, and avoidance of unnecessary conversions. Commitment to technical refinement, evidence-based validation, and structured skill acquisition strengthens OPCAB surgery’s viability.

Looking forward, the future of OPCAB surgery depends on overcoming institutional barriers, embracing emerging technologies, and driving innovation. Standardized training, refined selection criteria, and integration of intraoperative imaging, robotics, and artificial intelligence will enhance reproducibility and success. Accreditation pathways and multidisciplinary collaboration are essential for ensuring OPCAB remains safe, reliable, and accessible across diverse health care settings.

Through sustained excellence, innovation, and evidence-based practice, OPCAB can fulfill its potential—delivering individualized, effective coronary revascularization and advancing the field of cardiac surgery.
