# Supplementary material for: Ten Commandments of Off-Pump Coronary Artery Bypass Surgery
Source: Ann Thorac Surg Short Rep. 2025 Oct 23;4(2):579–84. doi: 10.1016/j.atssr.2025.09.027 (PMC13245340; doi:10.1016/j.atssr.2025.09.027)
Supplement: Supplementary Material [file mmc6.docx]

**Supplemental File**

**Technical Aspects of Off-Pump Coronary Artery Bypass Grafting**

**Fundamental Principles**

Off-pump coronary artery bypass grafting (OPCAB) demands the same level of precision as traditional on-pump procedures. The absence of cardiopulmonary bypass must never compromise the quality of anastomoses. Surgeons should maintain consistent suturing techniques across both modalities. Following a midline skin incision, a full median sternotomy is performed. The left internal thoracic artery (ITA) is harvested under direct visualization using a lifting retractor. Bilateral ITA harvesting—either skeletonized or pedicled—is recommended for suitable candidates. The radial artery and saphenous vein are retrieved endoscopically.

Monofilament 8-0 sutures are used for all distal anastomoses, with meticulous attention to each stitch. The needle should enter the graft from outward into the lumen and the coronary artery from the lumen outward. Clear visualization of the intimal layers is essential to avoid posterior wall injury. A misted blower delivering pH-balanced fluid with minimal CO₂ pressure is routinely employed to enhance visibility.

Each anastomosis typically involves 12 evenly placed stitches (akin to the numbers on the dial of a clock) to ensure optimal geometry.

**Cardiac Exposure and Positioning**

***Enhancing Target Visibility***

A broad inverted-T pericardial incision is made, followed by division of the diaphragm at its attachment to the right xiphoid process. This maneuver elevates the right sternal border, aided by rolled towels beneath the retractor. Bilateral pleural spaces should be opened when harvesting both ITAs or when rightward cardiac displacement is required due to cardiomegaly or low ejection fraction.

Optimal heart positioning is achieved using superficial pericardial traction sutures, a deep posterior stitch, and a suction-based positioning device. Superficial sutures are placed 2 cm from the pericardial edge to allow selective retraction. A deep stitch is placed in the posterior pericardium, two-thirds of the way between the inferior vena cava and the left inferior pulmonary vein, near the pericardial reflection over the posterior left atrium. This suture is protected with a soft rubber sleeve to prevent epicardial injury.

The deep stitch is retracted either caudally to elevate the heart’s base or laterally to expose anterior and lateral surfaces. Cardiac displacement should be gradual and gentle. The heart tolerates rotation well but is sensitive to compression, particularly of the right ventricular outflow tract. Simultaneous bilateral pericardial retraction should be avoided to prevent reduced caval inflow and hemodynamic compromise.

***Suction-Based Cardiac Positioner***

This device can be applied to any cardiac surface. When elevating the apex to expose the inferior wall, avoid excessive cranial bending to prevent outflow tract compression. In patients with ischemic mitral regurgitation, extra care is needed during elevation. The device should not be placed over epicardial fat fissures, which may disrupt suction, nor too close to coronary targets to avoid interference with the stabilizer.

***Coronary Stabilizer***

The stabilizer’s dual suction pods are independently adjustable to conform to the heart’s surface. Before locking the device, observe the full vertical motion of the target vessel during the cardiac cycle and apply the stabilizer at the midpoint of this excursion. Excessive pressure should be avoided, as it may paradoxically increase vessel movement and impair stability. The device functions via suction—not compression.

**Intracoronary Shunting**

There is no definitive evidence favoring or opposing shunt use. Soft silicone shunts may reduce ischemia, prevent reperfusion injury, and protect the posterior coronary wall. Some surgeons prefer a non-shunt approach for better visualization, but shunts may be necessary in cases of hemodynamic instability or poor visibility due to collateral flow.

Signs of ischemic arrhythmia during grafting warrant immediate shunt placement. In RCA procedures, shunting may prevent bradycardia and hypotension, serving as an alternative to pacing. For trainees, shunts offer added protection against inadvertent suture placement.

**Anastomotic Sequence and Vessel Exposure**

***Strategic Grafting Order***

Grafts are placed in a sequence that gradually increases cardiac displacement: anterior wall vessels (left anterior descending [LAD], diagonal, ramus intermedius), followed by inferior wall targets (right coronary artery [RCA], posterior descending artery [PDA]), and finally lateral wall branches (obtuse marginal, posterolateral). This progression improves tolerance as revascularization proceeds.

*Anterior Wall*

The table remains flat or slightly head-down. The deep pericardial stitch is pulled toward the patient’s left hip, elevating the heart with minimal hemodynamic impact. The LAD is centered in the stabilizer, temporarily occluded using a silastic loop, Prolene suture with snugger, or bulldog clamp. Arteriotomy and shunt placement follow, aided by a CO₂ blower. Diagonal artery grafting uses the same approach. Intramyocardial ramus intermedius may require vertical heart positioning near the base.

*Inferior Wall*

For PDA exposure, the table is placed in steep Trendelenburg (20°) and rotated 10–20° toward the surgeon. Swabs support apical elevation, while the deep pericardial stitch is pulled caudally and secured left of midline. The stabilizer foot is directed downward to secure the PDA.

Temporary RCA occlusion proximal to bifurcation is necessary but may compromise AV node perfusion. Epicardial pacing wires are placed to prevent bradycardia and distension. For RCA grafting, the table is flattened, allowing the heart to shift leftward naturally. The stabilizer is positioned per surgeon preference.

*Lateral Wall*

To expose obtuse marginal and posterolateral branches, the table is kept in 20° Trendelenburg and rotated 10–20° to the right. Gravity shifts the heart rightward and the apex anteriorly, enhancing venous return and promoting counterclockwise rotation. Pericardial sutures on the right are removed to allow greater displacement. In select cases, opening the right pleura adds mobility without compressing the right ventricle.

Posterior pericardial sutures should be placed midway between the left inferior pulmonary vein and the inferior vena cava. Displacement must be slow and deliberate, using suction devices, traction sutures, table adjustments, and gravity. The coronary stabilizer is placed on the right side of the retractor for optimal lateral wall access.

**Conclusion**

Successful OPCAB requires a structured approach: precise pericardial release, controlled cardiac displacement, and strategic graft sequencing. This methodology ensures optimal vessel exposure, minimizes myocardial stress, and enhances long-term graft patency. Mastery of these techniques supports stable hemodynamics and effective myocardial revascularization.

**Supplementary video 1.**

**Supplementary video 2.**

**Supplementary video 3.**

**Supplementary video 4.**

**Supplementary video 5.**

**Supplementary Table 1.** Recommended grafting sequence and exposure strategy in OPCAB, aligned with progressive cardiac displacement, myocardial tolerance, and anatomical presentation

| \| **Step** \| **Target Vessel** \| **Suggested Graft** \| **Positioning Strategy** \| **Stabilization Technique** \| **Exposure & Retraction Notes** \| \| --- \| --- \| --- \| --- \| --- \| --- \| \| 1 \| Left Anterior Descending (LAD) \| Left Internal Mammary Artery (LIMA) or Right Internal Mammary Artery (RIMA) \| Supine or slight head-down; minimal displacement with a rolled swab under the heart \| Suction stabilizer \| Traction suture pulled toward left hip; LAD centered in stabilizer; temporary occlusion with loop/snugger/clamp; CO₂ blower mister and intracoronary shunt used \| \| 2 \| Diagonal Branch \| LIMA or Radial Artery (RA) or Saphenous Vein Graft (SVG) \| Supine, slight lateral tilt \| Suction stabilizer \| Similar exposure as LAD; CO₂ blower mister and intracoronary shunt used \| \| 3 \| Ramus Intermedius \| RA or SVG \| Supine, vertical heart positioning if intramyocardial \| Suction stabilizer \| Grafting near heart base; may require deeper traction and apex elevation; CO₂ blower mister and intracoronary shunt used \| \| 4 \| Posterior Descending Artery (PDA) \| SVG or RA \| Steep Trendelenburg (20°), table rotated 10–20° toward surgeon \| Apical suction + Stabilizer per surgeon preference \| Swabs secure apex; snare pulled caudally and left of midline; stabilizer foot directed downward; pacing wires placed to prevent AV node compromise; CO₂ blower mister and intracoronary shunt used \| \| 5 \| Obtuse Marginal / Posterolateral \| RA or SVG \| Trendelenburg (20°), table rotated 10–20° rightward \| Stabilizer on right side of retractor \| Gravity shifts heart rightward and apex; anteriorly; posterior traction sutures placed between LIPV and IVC; heart rotated incrementally without compression; right pleural opening optional for enhanced exposure and translocation; CO₂ blower mister and intracoronary shunt used \| |
| --- | --- | --- | --- | --- | --- | --- | --- | --- | --- | --- | --- | --- | --- | --- | --- | --- | --- | --- | --- | --- | --- | --- | --- | --- | --- | --- | --- | --- | --- | --- | --- | --- | --- | --- | --- | --- |

AV = atrioventricular; LIPV = left inferior pulmonary vein; IVC = inferior vena cava; OPCAB = off-pump coronary artery bypass
